# PReS-FINAL-2341: Perturbed homeostasis of FOXP3+ regulatory T cells and STAT1 signaling in SLE patients with childhood and adult onset of disease

**DOI:** 10.1186/1546-0096-11-S2-P331

**Published:** 2013-12-05

**Authors:** M Holcar, A Goropevšek, N Semolič, A Pahor, T Avčin

**Affiliations:** 1 Department of Allergology, Rheumatology and Clinical Immunology, University Children's Hospital, University Medical Centre Ljubljana, Ljubljana, Slovenia; 2 Department for Laboratory Diagnostics, University Medical Centre Maribor, Maribor, Slovenia; 3 Division of Internal Medicine, Department of Rheumatology, University Medical Centre Maribor, Maribor, Slovenia

## Introduction

Dysregulation of many inflammatory cytokines, utilizing STAT signaling pathways, has been found as important contributor in initiation, progression and maintenance of inflammation in patients with Systemic Lupus Erythematosus (SLE).

FoxP3^+^CD4^+ ^regulatory T cells (Tregs) are important mediators of peripheral immune tolerance and their perturbed homeostasis, including expansion of CD45RA^-^FoxP3^lo ^non-Treg subpopulation was reported in adult patients with SLE. Type I and II interferons (IFN I and IFN II), which are implicated in SLE pathogenesis, were shown to perturb Treg homeostasis. Many IFN regulated genes are dependent on STAT1 for optimal transcription, and STAT1 protein expression is under control of IFNs.

## Objectives

In this study we focused on FoxP3 expressing T cells subsets and IFN linked aberrances in expression of STAT1 in T-cells from childhood-onset SLE patients.

## Methods

The pediatric study population consisted of 13 patients (12 female and 1 male) with childhood onset SLE, mean age 16.9 years, from which we obtained all together 25 samples at their routine 3-month checkup. 20 healthy blood donors (all female, mean age 17.0 years) were used as controls. Another study population consisted of 34 patients with adult onset SLE (31 female and 3 male) with mean age at the time of diagnosis 33.0 years.

Flow cytometric analysis of T cell STAT1 protein expression was performed after surface and intracellular staining in EDTA whole blood. In addition, expression of FoxP3 and CD45RA on CD4^+^T cells was studied, which enabled delineation of FoxP3 expressing cells into recently described subsets, including CD45RA^-^FoxP3^lo ^non-Tregs. Cells were analysed using FACSCanto II and LSRII Flow Cytometers (BD Biosciences) and subsequent analysis using FlowJo software (Tree Star). Two-tailed Mann-Whitney test was used to evaluate differences between groups.

## Results

SLE T cells showed significantly higher STAT1 protein expression (p < 0.0001) than those of healthy controls, which were however not significantly different between pediatric and adult patients. This indicates strong IFN signature and suggest mechanism of inflammation self-maintenance utilizing the JAK-STAT signaling pathway also in pediatric SLE patients. Consistent with this we also found significantly higher frequencies of CD45RA^-^FoxP3^lo ^T cells (p < 0.0001), which were reported to secrete IFN-gamma, in patients with pediatric onset SLE.

## Conclusion

Findings support the role of IFNs and aberrant STAT signaling, that may drive autoimmunity in SLE and functional deviation of Tregs that can also contribute to the pathogenesis of this disease. Our data suggest common T cell dysfunctions in patients with both childhood and adult onset SLE, suggesting that differences in clinical manifestations and disease severity between children and adults may be consequence of different developmental state of affected organs and not novel (specific) etiopathogenesis.

## Disclosure of interest

None declared.

